# In Situ FT-IR Characterization of CuZnZr/Ferrierite Hybrid Catalysts for One-Pot CO_2_-to-DME Conversion

**DOI:** 10.3390/ma11112275

**Published:** 2018-11-14

**Authors:** Ivana Miletto, Enrico Catizzone, Giuseppe Bonura, Chiara Ivaldi, Massimo Migliori, Enrica Gianotti, Leonardo Marchese, Francesco Frusteri, Girolamo Giordano

**Affiliations:** 1 Dipartimento di Scienze ed Innovazione Tecnologica, Università del Piemonte Orientale “Amedeo Avogadro”, Viale Teresa Michel, 11. 15100 Alessandria, Italy; ivana.miletto@uniupo.it (I.M.); chiara.ivaldi@uniupo.it (C.I.); enrica.gianotti@uniupo.it (E.G.); leonardo.marchese@uniupo.it (L.M.); 2 Dipartimento di Ingegneria per l’Ambiente e il Territorio e Ingegneria Chimica, Università della Calabria, via P. Bucci, 87036 Rende, Italy; enrico.catizzone@unical.it (E.C.); ggiordaunical@yahoo.it (G.G.); 3 CNR-ITAE, Istituto di Tecnologie Avanzate per l’Energia “Nicola Giordano”, via S. Lucia sopra Contesse 5, 98126 Messina, Italy; giuseppe.bonura@itae.cnr.it (G.B.); francesco.frusteri@itae.cnr.it (F.F.)

**Keywords:** CO_2_ hydrogenation, dimethyl ether, Cu‒ZnO‒ZrO_2_, ferrierite zeolite, catalyst deactivation, zeolite Brønsted/Lewis acidity, FTIR

## Abstract

CO_2_ hydrogenation to dimethyl ether (DME) is a promising strategy to drive the current chemical industry towards a low-carbon scenario since DME can be used as an eco-friendly fuel as well as a platform molecule for chemical production. A Cu‒ZnO‒ZrO_2_/ferrierite (CZZ/FER) hybrid grain was recently proposed as a catalyst for CO_2_-to-DME one-pot conversion exhibiting high DME productivity thanks to the unique shape-selectivity offered by ferrierite zeolite. Nevertheless, such a catalyst deactivates but no direct evidence has been reported of activity loss over time. In this work, CZZ/FER catalysts with different acidity levels were characterized with the FTIR technique before and after reactions, aiming to give new insights about catalyst deactivation. Results show that activity loss can be related to both (i) copper particle sintering, which decreases CO_2_ activation towards methanol, and (ii) acidity loss due to H^+^/Cu^2+^ ion exchange.

## 1. Introduction

Dimethyl ether (DME), the simplest of the ethers, is receiving renewed attention since DME is considered a reliable alternative fuel for diesel engines thanks to its high cetane number, low ignition temperature and soot-free exhaust as well as it being a key intermediate compound for the production of olefins and gasoline-cut hydrocarbons [1,2,3,4,5]. 

Conventionally, DME is produced in gas phase in a two-step process whereby methanol is firstly produced from syngas over a Cu-based catalyst and DME is then produced by methanol dehydration over an acid catalyst in a second unit. γ-Al_2_O_3_ is the traditional industrial acid catalyst for the methanol dehydration thanks to its high DME selectivity. On the other hand, high reaction temperatures are necessary to promote alcohol conversion because the water molecules formed during the reaction suppress the catalytic activity of γ-Al_2_O_3_ [6,7,8]. DME can also be produced from syngas in a one-step process using a multi-functional catalyst. The one-step route is more efficient than the two-step route, mainly because of the thermodynamic advantages related to the equilibrium shift of simultaneous reactions (methanol dehydration to DME promotes the syngas conversion) and the lower overall processing cost. In addition, valorization and reusing of carbon dioxide is an important challenge in order to mitigate global warming. On this account, the synthesis of DME by total or partial replacement of CO with CO_2_ is receiving more and more attention [9,10,11,12]. 

A more active and water-resistant material needs to be developed for sustainable and reliable DME industrial production. Zeolites are being investigated as alternative acid catalysts to γ-Al_2_O_3_ and several works have revealed that the channel system, acidity and crystal morphology strongly affect the catalytic behaviour in terms of DME yield, deactivation and coke formation [13,14,15,16,17,18]. In particular, FER-type is considered a reliable zeolite catalyst for DME production via both methanol dehydration and CO_2_ hydrogenation due to its unique shape-selective structure that allows for obtaining high DME yield and inhibiting coke formation [19,20]. 

Physical mixture of Cu‒ZnO catalyst with zeolites is the simplest way to obtain a bi-functional catalyst for DME synthesis, even though other methods have been developed such as co-precipitation, impregnation, sedimentation, sol-gel and liquid-phase synthesis [21,22,23]. For instance, in 2015, Frusteri et al. [24] showed that multifunctional CuZnZr(CZZ)-MFI single grain prepared via co-precipitation of metal precursors over zeolite crystals enhanced the DME yield in comparison with a system prepared by conventional physical mixing between a Cu-based methanol catalyst and zeolite particles. The authors claimed that interaction between metals and acid sites is of paramount importance to obtain high DME productivity. Furthermore, Frusteri et al. [19,25] studied the effect of zeolite structure on the catalytic behaviour of a CZZ‒zeolite hybrid grain during one-pot CO_2_-to-DME, highlighting the superiority of ferrierite zeolite thanks to more efficient red-ox/acid cooperation to convert CO_2_ into methanol and rapidly dehydrate the alcohol into DME with a low production of by-products as carbon monoxide. The superiority of ferrierite over other zeolites was also confirmed by Prasad et al. in a syngas-to-DME process [15], confirming that the choice of acid catalyst is fundamental for DME production via both indirect (methanol dehydration) and direct (syngas/CO_2_-to-DME) synthesis [22,23]. Recent work by Bonura et al. [26] reports that the catalytic behaviour of CZZ‒ferrierite is strongly affected by the acidic properties of zeolites. In particular, the authors found that the higher the acidity of zeolite, the faster the deactivation rate of hybrid catalyst.

A deep characterization of the surface properties of a catalyst is of paramount importance in catalysis, since several aspects should be taken into account in order to rationalize the experimental results of catalytic tests. FT-IR is considered one the most promising techniques to study the surface properties of catalysts. In this work, the CZZ‒ferrierite hybrid catalysts used in [26] for one-pot CO_2_-to-DME synthesis are characterized via in situ FT-IR analysis by using different probe molecules (namely, acetonitrile and CO). The obtained characterization results are discussed in light of previously published catalytic data, aiming to better elucidate the role of surface properties of such materials in catalytic behaviour during the one-step CO_2_-to-DME process, especially in terms of activity and stability.

## 2. Experimental 

The CZZ‒FER samples investigated in this work are the same used by Bonura et al. in a recent work [26]. CuZnZr‒ferrierite samples are prepared via co-precipitation at room temperature of metal precursors (Cu/Zn/Zr molar ratio of 60/30/10) by oxalic acid at room temperature over ferrierite crystals (metals: zeolite weight ratio of 1:1) having different Si/Al, followed by calcination and reduction before reaction. Further details about sample synthesis, textural properties and catalytic tests in the CO_2_-to-DME one-pot conversion are reported in [26]. For the sake of clarity, the same sample labelling method used in [26] was adopted: CZZ‒FERx refers to CuZnZr‒ferrierite hybrid samples and x is the Si/Al ratio of zeolite (namely, 8, 30 and 60). As for zeolite samples, the label HFERx refers to pristine samples before metal deposition.

FTIR measurements were carried out using a Bruker Equinox 55 spectrometer (Billerica, MA, USA) equipped with a pyroelectric detector (DTGS type), operating in transmission (64 scans at 4 cm^−1^ resolution). 

Deuterated acetonitrile (d_3_-acetonitrile) vapour pressure was adsorbed at room temperature using specially designed quartz cells (DISA S.a.S, Milan, Italy) equipped with KBr windows and permanently connected to a vacuum line to perform adsorption–desorption in situ measurements. The samples, in the form of self-supported pellets, were preliminary outgassed at 623 K under vacuum conditions (residual pressure <10^−5^ mbar) for 1 h and then cooled to room temperature prior to the d3-acetonitrile adsorption experiments. d_3_-acetonitrile vapour was adsorbed at room temperature (r.t.) on the samples. After 30 min of contact, d_3_-acetonitrile was desorbed at r.t. (1 h outgassing). FTIR spectra were normalized with respect to the pellet density and, whenever specified, are reported in difference mode by subtracting the spectrum of the sample in a vacuum from the spectrum of the adsorbed molecules. 

The total number of accessible acid sites (N) was estimated using the Lambert–Beer law in the form A = εNρ, where A is the integrated area of the bands of the protonated species, ε is the molar extinction coefficient (cm mmol^−1^), N is the concentration of the vibrating species (mmol g^−1^), and ρ is the density of the disk (mass/area ratio of the pellet, mg cm^−2^). The quantification of the accessible sites was done on the spectra obtained upon outgassing at r.t. the adsorbed probe molecules and by using the following values as the molar extinction coefficients: d3-acetonitrile on Brønsted acid sites, band at ca. 2297 cm^−1^, ε = 2.05 cm mmol^−1^; d_3_-acetonitrile on Lewis sites, band at 2310–2325 cm^−1^, ε = 3.6 cm mmol^−1^; d3-acetonitrile on silanols, band at 2175 cm^−1^, ε = 0.74 cm mmol^−1^.

FTIR measurements of adsorbed CO were carried out on self-supported pellets preliminarily outgassed at 623 K for 1 h in vacuum and then the pellet was maintained at 623 K and exposed to a H_2_ partial pressure of 120 mbar for 1 h (the ferrierite samples without supported metals did not undergo the reduction step). After the treatment, H_2_ was pumped off and the system was cooled down to r.t. CO was then adsorbed at r.t. at a pressure of 70 mbar. After equilibration at r.t., the system was cooled down to the liquid nitrogen temperature (LN) in order to allow CO to interact with the acid sites present on the catalysts. Spectra were recorded, lowering the CO pressure from the maximum coverage to a residual pressure <10^−5^ mbar.

## 3. Results and Discussion

### 3.1. FTIR Investigation of the Pristine HFER Catalysts

**Figure 1:**
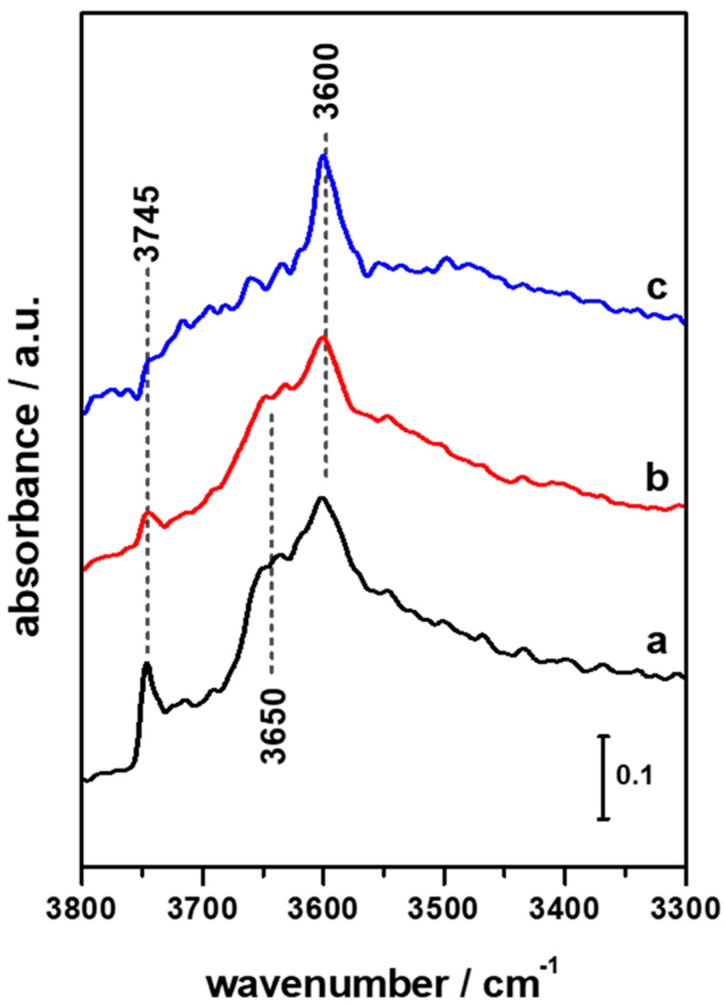
FTIR spectra of HFER8 (a, black curve), HFER30 (b, red curve) and HFER60 (c, blue curve) upon activation at 623K.

**Figure 2:**
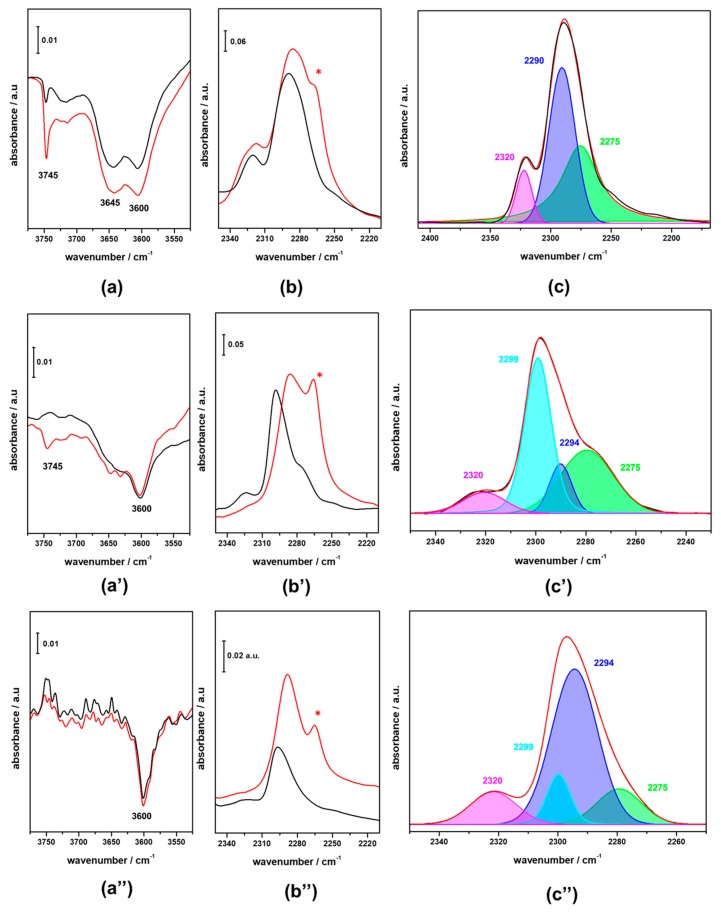
FTIR difference spectra of HFER catalysts at maximum d_3_-acetonitrile coverage and after desorption of the probe molecules at r.t. (red and black curves, respectively, in sections **a**, **a’**, **a”**, **b**, **b’**, **b”**). In section **c**, **c’** and **c”** the deconvolution of the irreversible fraction at r.t. is reported. (sections **a**, **b** and **c** refer to HFER8; sections **a’**, **b’** and **c’** refer to HFER30; sections **a”**, **b”** and **c”** refer to HFER60).

In Figure 1 FTIR spectra in the high-frequency region of pristine HFER catalysts upon activation at 623 K in vacuum are reported. The typical signal due to bridging OH groups (Brønsted groups) is present at ca. 3600 cm^−1^ in all three HFER catalysts. In HFER8 (curve a) and HFER30 (curve b), additional signals attributed to defective sites are also present; in particular, the shoulder around 3650 cm^−1^ can be ascribed to Al–OH groups, whilst the sharp signal at 3745 cm^−1^ indicates the presence of terminal Si–OH groups. These signals are barely visible in the case of the higher Si/Al ratio catalyst, HFER60 (curve c), and their intensity increases along with the decrease in the Si/Al ratio, indicating that higher Al content leads to a higher concentration of silanol groups as a result of the increased defectivity of the zeolite framework. In order to probe the accessibility of such acid sites in the as-prepared HFER catalysts and after the deposition of Cu/Zn/Zr species, adsorption of d3-acetonitrile was performed. As is widely known, acetonitrile is a weak base that interacts with acid sites through the nitrogen lone pair of the C≡N group; fundamental νs(CD_3_) and νas(CD_3_) of d_3_-acetonitrile (ca. 2114 and 2250 cm^−1^, respectively) are not significantly altered after acetonitrile adsorption on acid sites. On the contrary, the strength of the probe molecule bonding to acid sites strongly affects the frequency of the stretching mode νC≡N; the stronger the acid site, the higher the shift of the signal towards higher frequencies. In this respect, strong Lewis sites originating from aluminium exhibit a band at 2320–2330 cm^−1^, Brønsted acid sites in high silica zeolites are responsible for a band at 2294–2299 cm^−1^, and weaker interactions with terminal Si–OH groups yield a signal at 2275–2280 cm^−1^, whereas the contribution of physisorbed acetonitrile can be found at ca. 2265–2261 cm^−1^. In Figure 2 the FTIR difference spectra of HFER catalysts at maximum d_3_-acetonitrile coverage (red curves) and after desorption of the probe molecule at r.t. (black curves) are reported. The negative peaks in the high-frequency region of HFER8 spectra (section a) evidences that both Brønsted groups and defective sites are interacting with d_3_-acetonitrile at maximum coverage (red curve); in the low-frequency region (section b) the corresponding typical signal of the νC≡N of d_3_-acetonitrile molecules interacting with acid sites of different strength is present. 

The broad signal at ca. 2320 cm^−1^ is due to probe molecules adsorbed on extra framework aluminium sites, whilst the intense asymmetric band at ca. 2280 cm^−1^ arise from the overlapping of the contribution from d_3_-acetonitrile on Brønsted acid sites (expected at ca. 2299 cm^−1^) and on Si–OH groups (expected at ca. 2275 cm^−1^); the shoulder present at lower frequency (2261 cm^−1^) and labelled with an asterisk is due to physisorbed d_3_-acetonitrile. After desorption at r.t. (Figure 2a,b, black curves), most of the species remains in interaction with the probe molecule, apart from a fraction of silanols that experienced a more labile interaction.

FTIR difference spectra of HFER30 upon interaction with d_3_-acetonitrile at maximum coverage and after desorption of the probe molecule at r.t. (Figure 2, section a’ and b’) are dominated by the intense signal of the νC≡N of molecules interacting with Brønsted acid sites (2299 cm^−1^), thus confirming the lower defectivity of this sample with respect to the HFER8 catalyst. The small contribution of molecules interacting with Si–OH, visible in the spectra registered at maximum coverage, readily disappeared after desorption at r.t. In the case of the HFER60 catalyst (Figure 2, section a” and b”) the difference spectra, in the high-frequency region, at maximum coverage and after desorption of the probe molecule at r.t., are almost the same, indicating that the probe molecule experiences a strong interaction with Brønsted acid sites. In the low-frequency region (section b” of Figure 2) the desorption of probe molecules at r.t. causes the disappearance of the contribution from physisorbed d_3_-acetonitrile, as expected, leaving the signal almost unaltered due to νC≡N of molecules interacting with Brønsted acid sites, and evidenced the presence of a small number of molecules interacting with the extra framework aluminium species and Si–OH (signals at 2320 and 2275 cm^−1^, respectively). On the basis of the second derivative mode of the spectra, a deconvolution procedure was run that yielded the deconvolution patterns reported in sections c, c’ and c” of Figure 2. It is worth noting that, in the case of HFER30 and HFER60, the second derivative mode highlighted two contributions at 2299 and 2294 cm^−1^ instead of the single contribution at 2290 cm^−1^, related to the νC≡N of molecules interacting with Brønsted acid sites. This finding could be explained on the basis of a heterogeneous distribution of Brønsted acid sites in the HFER30 and HFER60 framework [27,28].

### 3.2. FTIR Investigation of the Fresh CZZ‒FER Catalysts

**Figure 3:**
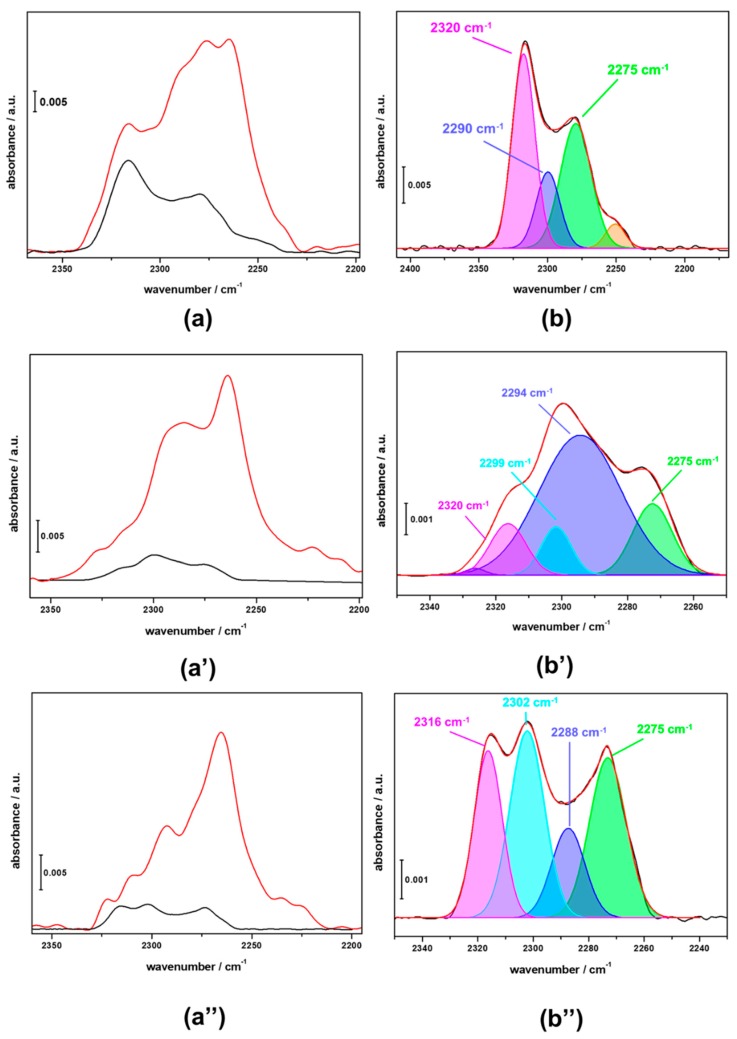
FTIR difference spectra of CZZFER catalysts at maximum d_3_-acetonitrile coverage and after desorption of the probe molecules at r.t. (red and black curves, respectively, in sections **a**, **a’**, **a”**). In sections **b**, **b’** and **b”** the deconvolution of the irreversible fraction at r.t. is reported. (Sections **a** and **b** refer to CZZFER8; sections **a’** and **b’** refer to CZZFER30; sections **a”** and **b”** refer to CZZFER60).

The FTIR investigation of the CZZFER8, CZZFER30 and CZZFER60 catalysts, obtained upon the deposition of copper, zirconium and zinc species over HFER8, HFER30 and HFER60 catalysts, was made difficult by the low transparency of the samples; in particular, the dark colour arising from the presence of metal species as well as the high scattering of the materials caused a lack of information from the high-frequency region of the spectra. Nevertheless, thanks to the good transparency in the low-frequency region, useful information about the nature and accessibility of acid sites in the CZZFER catalysts was obtained. In Figure 3 the FTIR difference spectra of CZZFER catalysts at maximum d3-acetonitrile coverage (red curves) and after desorption of the probe molecule at r.t. (black curves) are reported (sections a, a’ and a”), along with the deconvolution patterns obtained on the spectra upon r.t. outgassing of the probe molecule (sections b, b’ and b”). By monitoring the frequency region of the νC≡N, it is clearly seen that the spectra registered at the maximum d3-acetonitrile coverage look quite complex due to the overlapping of several contributions. In fact, d3-acetonitrile can interact, on the unreduced samples, not only with acid sites of the HFER support, but also with Cu^+^ and Cu^2+^ species, as well as with surface defects of ZrO_2_ and ZnO nanoparticles. It has been reported [29] that interaction of d_3_-acetonitrile with Cu^+^ and Cu^2+^ sites is evidenced by the presence of weak bands at 2297 and 2300–2310 cm^–1^, respectively; these complexes are stable only at high partial pressures of d_3_-acetonitrile and evacuation of the FTIR cell results in the disappearance of such contributions. As a consequence, the analysis of FTIR spectra after desorption of the probe molecule at r.t. allows for the identification of the stronger Lewis and Brønsted acid sites, as well as defective Si–OH, interacting with d_3_-acetonitrile. In the case of CZZFER8, the spectrum is dominated by the strong contribution at 2320 cm^−1^ due to Lewis acid sites and by the signal at 2275 cm^−1^, ascribable to terminal Si–OH groups. No Brønsted acid sites were identified, but the deconvolution of the spectrum showed a small contribution at 2290 cm^−1^, corresponding to the d_3_-acetonitrile in interaction with these sites. In the case of the CZZFER30 sample, despite the low intensity of the spectra, it is still possible to identify the signals due to Brønsted acid sites, although the relative intensities of the different contributions are significantly changed with respect to the parent HFER30 catalyst. Similar behaviour was detected in the CZZFER60 sample. Table 1 shows the concentration of accessible acid sites in both the parent HFER and the hybrid CZZFER catalysts.

It is clear that the co-precipitation of copper, zirconium and zinc species led to the formation of nanosystems that partially occluded the microporous network of CZZFER hybrid catalysts, so that a very small fraction of Brønsted acid sites, which are preferentially located within the zeolite channels, are accessible to d_3_-acetonitrile. A higher degree of accessibility was retained in the case of extra framework aluminium species and terminal Si–OH groups, indicating their different location within the zeolite framework. Furthermore, the decrease of Brønsted acid sites after co-precipitation of metal precursors may also be related to the partial exchange of zeolite protons by Cu^2+^ species, as reported by García-Trenco et al. [30,31]. In particular, our measurements showed that, in the case of FER8, after co-precipitation the number of Lewis sites increased from about 0.15 mmol/g_zeolite_ to about 0.24 mmol/g_zeolite_, highlighting the creation of Lewis sites probably due to ion exchange.

FTIR measurements of adsorbed CO were carried out on CZZFER systems after activation and reduction treatment in order to probe the nature of the Cu/Zn/Zr catalytic sites. CO was adsorbed at r.t. at a pressure of 70 mbar; after equilibration at r.t., the system was cooled down to the LN temperature, thus allowing CO to interact with the acid sites present on the parent FER framework (Brønsted and Lewis acid sites and defects). As a comparison, the FTIR measurements of adsorbed CO at r.t. and at LN temperature were also carried out on the parent FER samples; the results are reported in the Supplementary Materials (see Figure S1).

**Figure 4:**
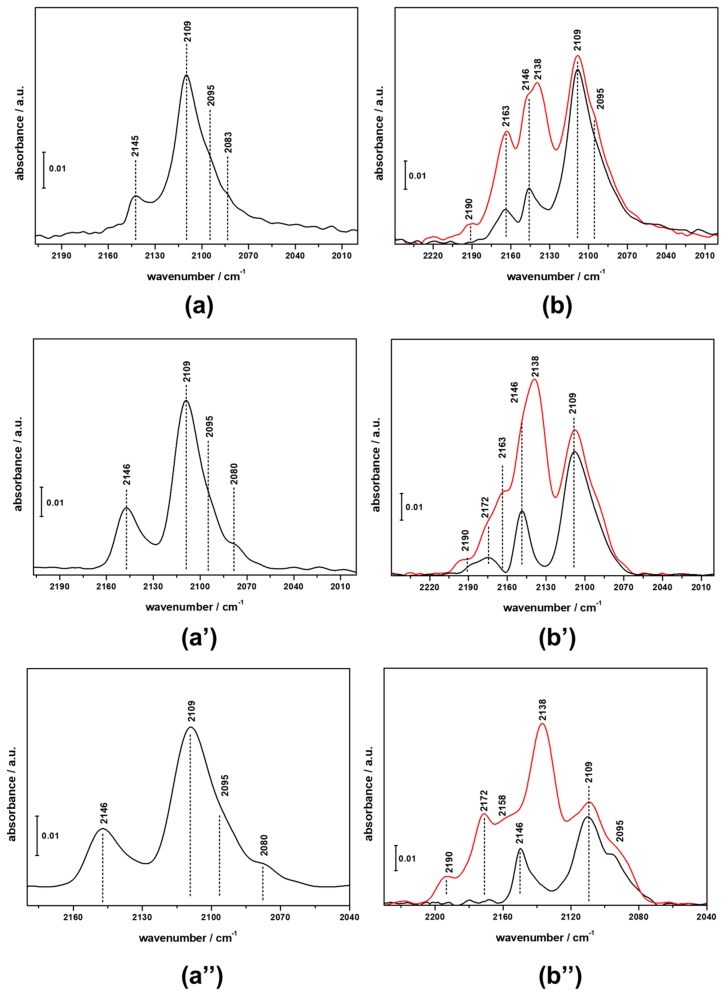
FTIR difference spectra of CO adsorbed on CZZFER catalysts at room temperature (sections **a**, **a’** and **a”**) and at LN temperature (sections **b**, **b’** and **b”**), at maximum coverage (red spectra) and after outgassing until the disappearance of the CO liquid-like signal at 2138 cm^−1^. (sections **a** and **b** refer to CZZFER8; sections **a’** and **b’** refer to CZZFER30; sections **a”** and **b”** refer to CZZFER60).

CO adsorption at r.t. on CZZFER8 samples (Figure 4, section a) evidenced an adsorption pattern in the 2130–2050 cm^−1^ range, which is typical of linear bound CO molecules on different Cu sites with a prevailing metal character [32,33]. In particular, the spectrum is dominated by a maximum at 2108 cm^−1^, with small shoulders around 2095 and 2083 cm^−1^ that can be ascribed to CO linearly bound to copper particles. In fact, for low-index surfaces (i.e., Cu(111), Cu(110) and Cu(100) planes of copper) absorbance maxima for adsorbed CO were reported in the literature to be between 2070 and 2095 cm^−1^; conversely, the main maxima for stepped surfaces (i.e., Cu(211), Cu(221) and Cu(532)) were reported to be shifted to higher frequencies, between 2095 and 2110 cm^−1^ [34]. 

Moreover, another contribution is visible at higher frequencies (2145 cm^−1^), which can be ascribed either to CO adsorbed on large copper particles [35] or to CO adsorbed on Cu^+^ sites [36]. When the temperature of the system is reduced to LN temperature, new features arise due to the interaction of carbon monoxide with the acid sites present in the parent CZZ framework as well as with the ZnO and ZrO_2_ and their defective sites. At high CO coverage, besides the signal at 2138 cm^−1^, which is due to liquid-like CO, two new components were detected. The intense and broad signal at 2164 cm^−1^ arises from the overlapping of the stretching signals of CO interacting with terminal Si–OH groups (expected at 2158 cm^−1^) and with Brønsted acid sites (expected at about 2172 cm^−1^). Furthermore, the small feature at 2190 cm^−1^ can be ascribed to CO in interaction with extra framework aluminium sites. It is worth noting that, at LN temperature, signals arising from CO molecules linearly adsorbed on the OH defect of ZnO and ZrO_2_ contribute to the signals at 2146 cm^−1^ and 2156 cm^−1^, respectively [37].

The same components reported for CZZFER8 were identified upon interaction of CO with CZZFER30 and CZZFER60 catalysts at r.t. Slight differences arise when the system is cooled down to LN temperature; in fact, due to the low concentration of silanol defects in the HFER30 and HFER60 with respect to the HFER8 sample, the typical absorption due to CO adsorbed on Brønsted acid sites and on silanols is well resolved and located at 2174 and 2158 cm^−1^, respectively. It is clear that the Si/Al ratio in the parent HFER does not significantly influence the nature or distribution of catalytic sites upon co-precipitation of Cu/Zn/Zr species. The IR adsorption lines at about 2160 cm^−1^ and 2190 cm^−1^ may also be related to Ni‒CO complexes [38,39], and this aspect was investigated since the transfer lines of the reactor unit are made of stainless steel (a good source of nickel). In order to check Ni contamination, atomic absorption measurements were carried out on exhausted catalysts (ContrAA^®^ 700—Analytic Jena AG, Jena, Germany). The results (not shown) clearly demonstrated the total absence of Ni species on exhausted samples; therefore, the observed bands are not associated with Ni‒CO complex formation.

### 3.3. FTIR Investigation of the Used CZZ‒FER Catalysts and Insights about Deactivation during CO_2_-to-DME One-Pot Conversion

**Figure 5:**
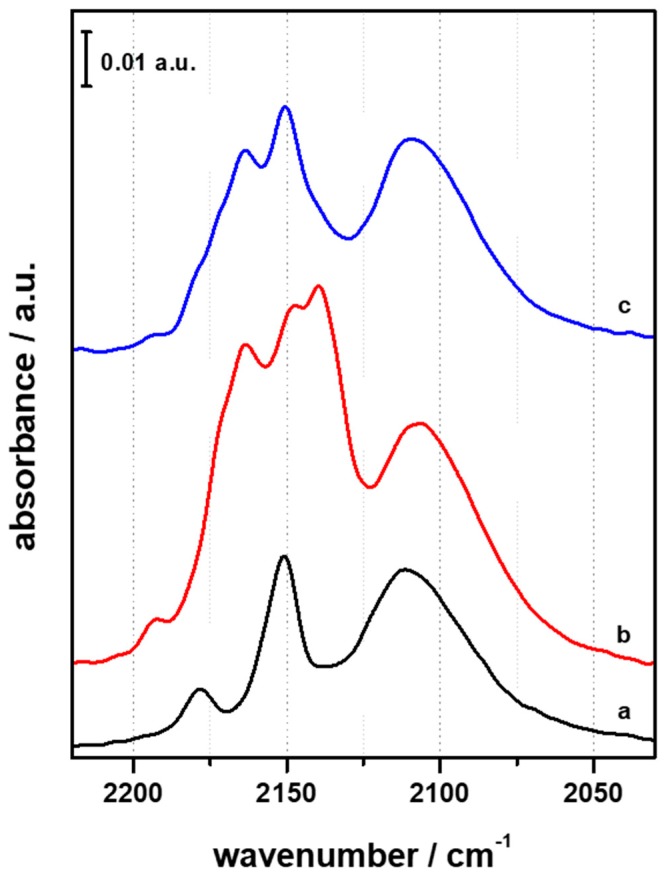
FTIR difference spectra of CO adsorbed at r.t. (a, black curve), at LN temperature (b, red curve) and at LN temperature after outgassing until the disappearance of the CO liquid-like signal at 2138 cm^−1^ (c, blue curve).

CZZ‒FER catalysts were characterized after the catalytic activity for 180 min during CO_2_-to-DME one-pot conversion at the following condition PR: 3.0 MPa, TR: 260 °C, GHSV: 2200 NL/kg_cat_/h, CO_2_/H_2_/N_2_: 3/9/1; more details are reported elsewhere [26]. After reaction, the “used” catalysts were cooled to r.t. under the prevailing reducing atmosphere of the reaction mixture and then pelletized for the FTIR measurement and treated at 533 K. CO adsorption at r.t. on the used CZZFER8 catalyst (Figure 5) evidenced an absorption pattern composed of the same signals already observed in the sample before catalysis, but with a significant difference in the relative intensities. The band in the 2130–20150 cm^−1^ range is still the most intense, but the relative intensity of the component at 2145 cm^−1^ is much higher than in the as-prepared sample; furthermore, a new signal arose at 2160 cm^−1^. This could indicate that, during the catalytic activity, evolution of the copper species occurs, which leads to an increase in the average size of the copper particles as well as to the adsorption of CO over forming cationic Cu^2+^ sites, as reported by Ordomsky et al. [40]. Similar behaviour was observed in the case of used CZZFER30 and CZZFER60 samples (see Figure S2 for details).

As reported by Bonura et al. [26], all the investigated catalysts exhibit deactivation during CO_2_-to-DME one-pot conversion. In particular, the authors show that at 260 °C the initial CO_2_ conversion was about 25% for all the catalysts. Furthermore, the authors even propose the following deactivation rate model, describing catalyst deactivation in terms of CO_2_ conversion:

$$X_{CO_{2}t}=X_{CO_{2}0}\cdot e^{-k_{deact}\cdot t}.$$

The deactivation coefficient k_deact_ was 5.96 × 10^−3^ h^−1^, 4.32 × 10^−3^ h^−1^ and 4.16 × 10^−3^ h^−1^ for CZZ‒FER8, CZZ‒FER30 and CZZ‒FER60, respectively. Consequently, after 180 min in time-on-stream the CO_2_ conversion was 20.9%, 21.9% and 22.1%, revealing a conversion drop of 20%, 14% and 13% for CZZ‒FER8, CZZ‒FER30 and CZZ‒FER60, respectively. The authors claim that the primary cause of catalyst deactivation is related to the progressive adsorption of water molecules over acid sites of zeolite, especially on more acidic catalysts, preventing methanol conversion. As reported in Section 2, for prior FTIR measurements the catalyst pellets were outgassed at 533 K with a residual pressure lower than 10^−5^ mbar, conditions in which the samples can be considered water-free. As reported in Table 1, after the reaction there is a reduction in the concentration of acid sites, especially for more acidic samples. In particular, the number of Brønsted sites usually decreases more than the number of Lewis sites. In fact, the decrease in acid sites concentration of CZZ‒FER8 and CZZ‒FER30, respectively, was 33% and 20% for Lewis sites, 75% and 21% for Brønsted sites and 54% and 71% for silanols, whilst no acidity changes were measured for CZZ‒FER60. Such results indicate that acid sites’ deactivation cannot be related to water adsorption since in this case no acidity changes should be observed after water removal. Confirming this assertion, Catizzone et al. have recently reported on the effect of water on the activity of FER-type zeolites during a methanol-to-DME dehydration step, showing that no activity changes were observed when water was co-fed with methanol and indicating that acid sites are completely available for DME formation in the presence of water [41]. Bonura et al. [26], after performing both TG-DSC and CHNS analysis of the “used” catalysts, found no coke traces; therefore, acidity reduction cannot be related to the adsorption of carbonaceous species on acid sites, as in other cases [42,43]. A plausible reason for the number of Brønsted acid sites decreasing after the reaction might be the migration of copper species from the zeolite surface to zeolite channels followed by ion exchange of copper ions with zeolite protons. García-Trenco et al. [30] found that Cu^2+^ species, as well as Zn^2+^ cations, occupy exchange positions in HZSM-5 zeolite, decreasing the number of Brønsted sites and generating Lewis sites in CuZnAl/HZSM-5 hybrid system during syngas-to-DME reaction and causing deactivation. 

Ordomsky et al. [40] found that during one-pot CO hydrogenation to dimethyl ether over the CuZnAl/HZSM5 system, Cu^2+^ species may exchange with proton sites of zeolites, decreasing both acidity and DME yield. Furthermore, the authors claim that copper migration may be hindered by silylation of the external surface of zeolites.

In our work, despite the difference in zeolite structure and the use of CO_2_ (instead of CO) as the carbon source, a similar phenomenon may be observed. In fact, the results suggest that the higher the ion exchange capacity (e.g., a higher Brønsted sites concentration), the higher may be the acidity loss due to H^+^/Cu^2+^ exchange. The presence of water formed by a reverse water gas shift reaction and methanol dehydration may play a role in such a phenomenon, promoting the formation of charged mobile copper clusters and migration inside of zeolite pores followed by ion exchange [44]. On the whole, on the basis of the obtained results, the catalyst deactivation of CZZ‒FER observed during CO_2_-to-DME one-pot conversion may be related to the deactivation of both metal and acid sites. Metal sites deactivate by copper particles’ sintering due to high temperature and high water content, while acid sites deactivation occurs by Cu^2+^ ion exchange with Brønsted sites of zeolites. The partial H^+^/Cu^2+^ exchange creates Lewis acid sites that do not significantly contribute in terms of methanol dehydration activity [30]. Metal sintering is the main causes of the slight deactivation of CZZ‒FER60 catalysts, whilst both mechanisms contribute to increasing the deactivation rate of CZZ‒FER8 and CZZ‒FER30 due to their higher ion-exchange capacity.

## 4. Conclusions

In this work, CuZnZr‒ferrierite catalysts showing high activity in the one-pot CO_2_-to-dimethyl ether process were characterized via FT-IR spectroscopy in order to give new insights into the catalytic behaviour exhibited by these materials. Both fresh and spent catalysts were characterized in terms of acidity via FT-IR by using both deuterated acetonitrile and CO as probe molecules. Bare ferrierite contains both Brønsted and Lewis acid sites, though Brønsted sites are mainly present in samples with lower aluminium content. Acidity is strongly reduced after precipitation of metals. Furthermore, some Brønsted sites may exchange with Cu^2+^, generating Lewis sites during the preparation of the catalyst. 

During the CO_2_-to-DME reaction all the catalysts exhibit deactivation in terms of both CO_2_ conversion and DME selectivity. Moreover, a decrease of acidity, with a more important effect on Brønsted acid sites, was measured. Neither carbonaceous compounds nor water, which may occupy acid sites, causing deactivation, were detected under the investigated conditions; therefore, a plausible reason for the number of Brønsted acid sites decreasing after reaction might be the migration of copper species from the zeolite surface to zeolite channels, followed by ion exchange of copper ions with zeolite protons. Such a phenomenon is more important for more acidic samples and may be promoted by the presence of water in the reaction system. 

## Figures and Tables

**Table 1:** Concentration of accessible acid sites in HFER and CZZFER catalysts.

Band Position and Assignment	Number of Acid Sites (mmol g^−1^)								
	HFER8	CZZFER8	CZZFER8 Used	HFER30	CZZFER30	CZZFER30 Used	HFER60	CZZFER60	CZZFER60 Used
2320 cm^−1^ Lewis sites	0.150	0.120	0.08	0.030	0.005	0.004	0.018	0.003	0.003
2290 cm^−1^ Brønsted sites	0.807	0.08	0.02	0.480	0.063	0.05	0.387	0.02	0.02
2275 cm^−1^ Silanols	0.930	0.520	0.24	0.250	0.035	0.01	0.190	0.01	0.01

## Supplementary Materials

The following are available online at http://www.mdpi.com/1996-1944/11/11/2275/s1, Figure S1: FTIR difference spectra in the CO stretching region of CO adsorbed at 80 K on HFER8 (a), HFER30 (b) and HFER60 (**c**) catalysts. Curve 1: Adsorption of 40 mbar of CO, Curves 1 to 20: decreasing CO doses up to 1 × 10^−4^ mbar., Figure S2: FTIR difference spectra of CO adsorbed on CZZFER30-used (a) and CZZFER60-used (b) at r.t. (black curve), at LN temperature (red curve) and at LN temperature after outgassing until the disappearance of the CO liquid-like signal at 2138 cm^−1^ (blue curve).
