# Supplementary material for: In Situ FT-IR Characterization of CuZnZr/Ferrierite Hybrid Catalysts for One-Pot CO2-to-DME Conversion
Source: Materials (Basel). 2018 Nov 14;11(11):2275. doi: 10.3390/ma11112275 (PMC6266444; doi:10.3390/ma11112275)
Supplement: Supplementary file 1 [file materials-11-02275-s001.pdf]

SUPPLEMENTARY MATERIALS

In Situ FT-IR Characterization of CuZnZr/Ferrierite Hybrid Catalysts for One-Pot CO_2-to-DME Conversion

Ivana Miletto [1], Enrico Catizzone [2], Giuseppe Bonura [3], Chiara Ivaldi [1], Massimo Migliori [2], Enrica Gianotti [1], Leonardo Marchese [1], Francesco Frusteri [3] and Girolamo Giordano [2,*]

[1] Dipartimento di Scienze ed Innovazione Tecnologica, Università del Piemonte Orientale "Amedeo Avogadro", Viale Teresa Michel, 11. 15100 Alessandria (Italy);

[2] Dipartimento di Ingegneria per l'Ambiente e il Territorio e Ingegneria Chimica, Università della Calabria, via P. Bucci, 87036, Rende (Italy);

[3] CNR-ITAE, Istituto di Tecnologie Avanzate per l'Energia "Nicola Giordano", via S. Lucia sopra Contesse 5, 98126, Messina (Italy)

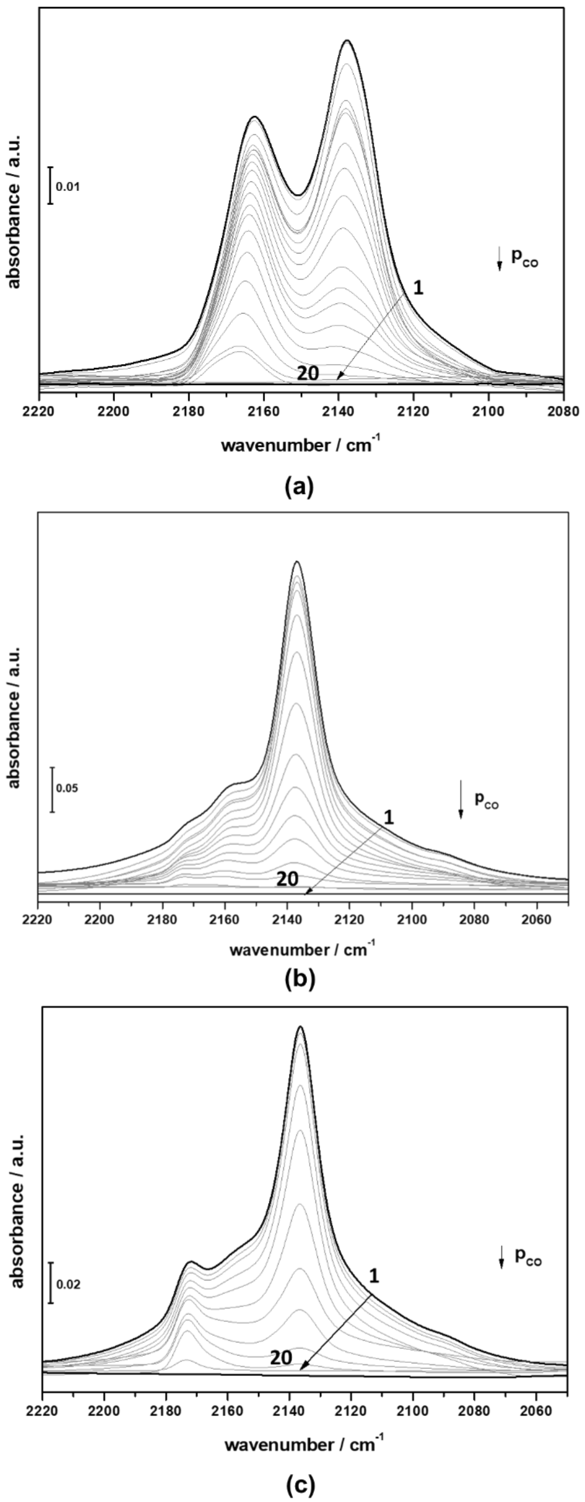

Figure S1 *FTIR difference spectra in the CO stretching region of CO adsorbed at 80 K on HFER8 (a), HFER30 (b) and HFER60 (c) catalysts. Curve 1: Adsorption of 40 mbar of CO, Curves 1 to 20: decreasing CO doses up to 1 x10^{-4} mbar*

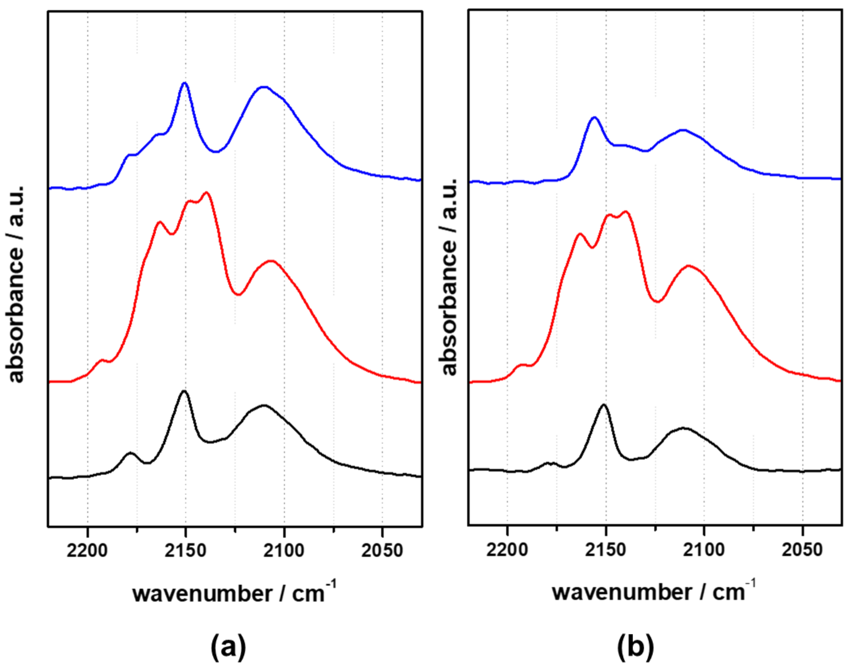

Figure S2 *FTIR difference spectra of CO adsorbed on CZZFER30-used (a) and CZZFER60-used (b) at r.t. (black curve), at LN temperature (red curve) and at LN temperature after outgassing until the disappearance of the CO liquid like signal at 2138* cm^{-1} *(blue curve).*
